# Coherence analysis of peripheral blood flow signals is a potential method for evaluating autonomic nervous system function

**DOI:** 10.3389/fphys.2025.1658174

**Published:** 2025-10-13

**Authors:** Qiuyue Lyu, Yuning Qin, Xin Wang, Qizhen Wang, Qi Liu, Na Tu, Yuhan Liu, Zixin Huo, Xiaojing Song, Shuyou Wang, Weibo Zhang, Xue Cao, Enshi Lu, Xiaoliang Zhao, Shuyong Jia, Liyun He, Guangjun Wang

**Affiliations:** ^1^ Institute of Acupuncture and Moxibustion, China Academy of Chinese Medical Sciences, Beijing, China; ^2^ Institute of Basic Research in Clinical Medicine, China Academy of Chinese Medical Sciences, Beijing, China; ^3^ Chinese Medicine Data Center, China Academy of Chinese Medicine, Beijing, China; ^4^ School of Acupuncture-Moxibustion and Tuina, Tianjin University of Traditional Chinese Medicine, Tianjin, China

**Keywords:** autonomic nervous system, coherence analysis, blood flow signal, heart rate variability, sympathetic nervous system, parasympathetic nervous system, gastrointestinal electricity

## Abstract

**Introduction:**

The autonomic nervous system (ANS) is crucial for maintaining homeostasis in the body and plays an important role in cardiovascular diseases. Although heart rate variability (HRV) is a commonly used non-invasive clinical tool to evaluate the function of ANS, it mainly reflects cardiac activity, and it is difficult to comprehensively capture the functional information of peripheral vascular regulation of ANS.

**Methods:**

This study explored the feasibility of using peripheral blood flow signals to evaluate the function of ANS. The ANS function of healthy subjects was artificially intervened by giving glucose solutions at different temperatures. Subsequently, the correlation between peripheral blood flow signals and HRV was further explored. Finally, the quantitative relationship was verified by using an independent dataset.

**Results:**

Coherence analysis shows that within a specific frequency band, the peak values of peripheral blood flow signals are significantly correlated with HRV.

**Discussion:**

This study shows that peripheral blood flow signals analysis provides a new non-invasive way to evaluate the function of ANS. This method not only complements the limitations of traditional HRV analysis, but also hopes to promote the construction of a more comprehensive and multi-dimensional ANS functional evaluation system.

## Graphical Abstract

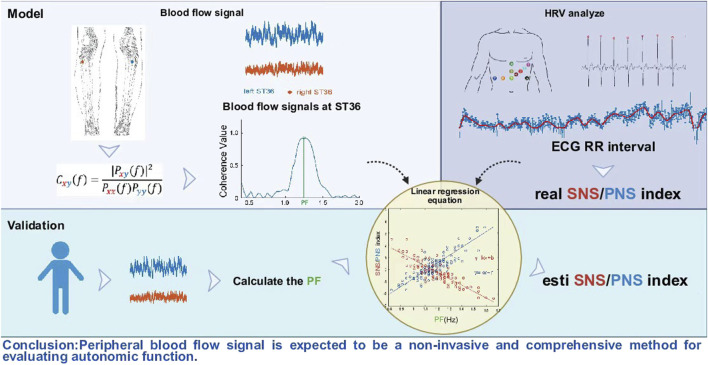

## 1 Introduction

The autonomic nervous system (ANS) mainly consists of two major branches: the sympathetic nervous system (SNS) and the parasympathetic nervous system (PNS) (Waxenbaum et al., 2019). It is composed of neurons that control various organ systems in the body and maintain homeostasis through many different chemicals and signals (Sztajzel, 2004). It controls involuntary processes related to the myocardium, smooth muscle, and exocrine and endocrine glands throughout the life cycle of the organism (Park, 2023), thereby regulating blood pressure, urination, defecation, and body temperature (McCorry, 2007). It is worth noting that clinical studies in the past 2 decades have revealed a significant association between the ANS and cardiovascular mortality, especially in patients with myocardial infarction and heart failure (Sztajzel, 2004). An imbalance of the ANS may lead to ventricular tachycardia and arrhythmia, which have become important causes of cardiovascular death (Franciosi et al., 2017; Goldberger et al., 2019). Therefore, evaluating the function of the ANS and clarifying the clinical diagnosis are highly important.

In recent years, heart rate variability (HRV) analysis has gradually become a core tool in this field due to its unique characterization ability of the autonomic nervous system. By quantifying the dynamic changes of the time interval between adjacent heart beats (Rajendra Acharya et al., 2006; Tiwari et al., 2021), HRV reveals the real-time regulation ability of ANS on the heart, especially the activity of SNS and PNS. However, this evaluation method still faces significant challenges: it is quite difficult to precisely quantify the independent contributions of each branch of ANS, and the interpretation of HRV analysis itself is complex and highly controversial. For instance, a large number of studies have observed that the low frequency (LF) component of HRV may simultaneously incorporate the combined influence of the sympathetic and parasympathetic nerves (Shaffer and Ginsberg, 2017). In contrast, blood vessels are almost exclusively innervated by the sympathetic nerve (Tucker et al., 2017),such as blood vessels located in the salivary glands, digestive tract, and reproductive organs. In this case, we can only talk about an increase or decrease in sympathetic nerve tone. In addition, an important feature of the sympathetic nervous system is that it can cause uneven changes in peripheral sympathetic nerve activity, thereby achieving selective regulation of local blood circulation (Rashid and Roatta, 2022). A typical example is temperature stimulation: both thermal exposure and cold exposure usually increase the overall level of sympathetic nerve activity and catecholamine concentration (Powers et al., 1982). However, the specific vascular effects of the two are diametrically opposite-heat stimulation mainly causes visceral vasoconstriction, while cold stimulation mainly causes skin vasoconstriction (Greaney et al., 2016).

Therefore, although HRV can indirectly reflect the impact of some SNS activities on the heart, it still falls short in precisely capturing the dynamic regulatory role of SNS in the broader systemic peripheral vascular system, especially the heterogeneous response patterns that may occur in SNS in different regions or different vascular.

Peripheral vascular system include arteries, arterioles and veins (CONRAD and GREEN, 1964). Previous study (Ipólito and Ferreira, 2013) have shown that sympathetic nerve stimulation can cause vasoconstriction, significantly affecting peripheral vascular circulation. For example, at the peripheral level, angiotensin II promotes neuronal transmission in the sympathetic ganglia, which is conducive to the release of norepinephrine from sympathetic nerve endings, acts on presynaptic receptors (Starke, 1977), and binds to α-adrenergic receptors on skin vascular smooth muscle, resulting in smooth muscle contraction and peripheral vasoconstriction. This neuro-vascular coupling mechanism indicates (Fan et al., 2023) that the dynamic changes of peripheral blood flow signals can reflect the intensity of SNS activities and provide other dimensions for the functional assessment of ANS. The ANS maintains homeostasis by regulating the function of organs such as those associated with the cardiovascular, respiratory, and digestive systems (Jänig, 2022), and the activity of these organs affects peripheral blood flow. Therefore, as a composite signal, the advantage of peripheral blood flow is that it carries information from various organs and tissues of the whole body, which can reflect the information of multiple physiological parameters and physical conditions at the same time (Green et al., 1944; Sparks Jr, 1999). For example, by analysing changes in blood flow velocity, the elasticity of blood vessels can be indirectly understood (Tao et al., 2004). Fluctuations in blood flow can reflect the stability of cardiac pumping function (Boselli et al., 2015).

Based on the above considerations, we believe that peripheral blood fluidity is dynamically regulated by ANS, and its dynamic process is essentially the result of ANS regulation. Then, it is theoretically feasible to evaluate the function of ANS through peripheral blood flow signals. Based on previous studies, we have noted that a 5% glucose solution, as an isotonic carrier, can eliminate the potential confounding effect of sodium ions on vagus nerve function. Simultaneously, the gastrointestinal tract expresses multiple temperature-sensitive TRP channels. TRPM8 is sensitive to temperatures below 25 °C, while TRPA1 is activated at temperatures below 17 °C (Jardín et al., 2017), and 30 °C, being close to the physiological stomach temperature, does not generate thermodynamic stress (O'Brien et al., 2024). Therefore, we adopted 4 °C and 10 °C as “cold stimuli” to attempt to activate the cold sensory pathways in the stomach and trigger ANS-specific regulation, while 30 °C was used as isothermal control to eliminate the interference of temperature variables on the study of glucose metabolism. The ANS function of healthy subjects was artificially interfered with by drinking glucose at different temperatures. Then, on the one hand, explore the correlation between peripheral blood flow signals and the function of ANS; On the other hand, by using the linear regression equation, the quantitative relationship between peripheral blood flow signals and ANS was constructed. This provided a new perspective for non-invasive assessment of ANS and complemented the traditional HRV assessment methods, which is expected to establish a more comprehensive assessment system.

## 2 Methods

### 2.1 Ethics approval

This study was approved by the Institutional Research Ethics Boards of Acupuncture & Moxibustion, China Academy of Chinese Medical Sciences. In accordance with the Declaration of Helsinki (Cantín, 2013), each subject provided informed consent and had an adequate understanding of the procedure and purpose of this study.

### 2.2 Subject inclusion and exclusion criteria

The inclusion criteria were good health and an age between 18 and 60 years. The exclusion criteria were the presence of diseases affecting cardiovascular or autonomic regulation; the administration of any medication affecting cardiovascular or autonomic regulation; and pregnancy or menstruation at the time of testing.

### 2.3 Participants and design

This study employed a single-blind design and included a total of 60 healthy subjects, who were randomly assigned to three temperature intervention groups at 4 °C, 10 °C, and 30 °C in a 1:1:1 ratio.

**FIGURE 1:**
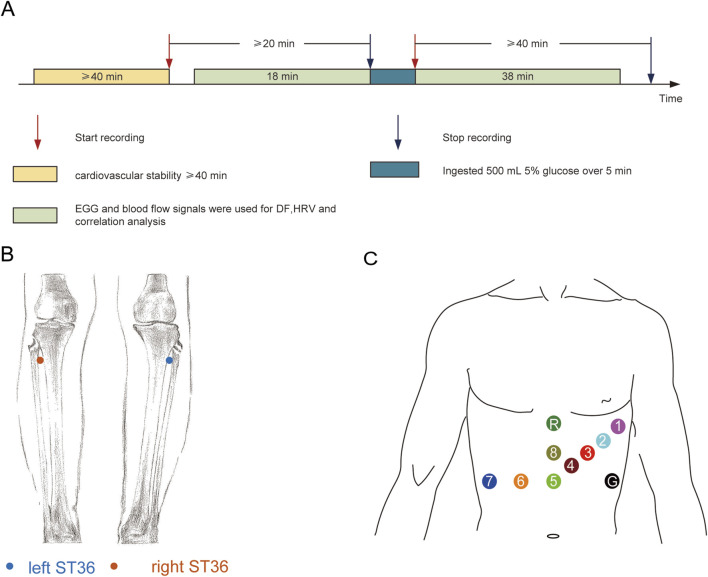
Experimental design and signal recording location: **(A)** Experimental design; **(B)** Recording location of blood perfusion signals at ST36 on both sides; **(C)** Location of the skin electrode for EGG recording.

The subjects were asked to abstain from consuming alcohol, tea, and coffee for at least 24 h prior to the test. Fast for more than 2 h before the experiment. A small amount of water is allowed. Arrive at the laboratory at the designated time and have the same meal. All the subjects completed the measurements and were included in the statistical analysis. The experimental design and signal recording locations are shown in Figure 1. All the experiments were conducted in a quiet, temperature-controlled (24 °C–26 °C) laboratory. After entering the laboratory, the subjects were asked to empty their bladders. Following a period of cardiovascular stability (40 min), a baseline recording was taken for at least 18 min. Then, over a 5–min period, the test subjects in the different groups ingested 500 mL of 4 °C, 10 °C, or 30 °C glucose, and the electrogastrogram (EGG) and blood perfusion were monitored for at least 38 min (as shown in Figure 1A). The recording location of blood flow signals in both lower limbs are shown in Figure 1B.

### 2.4 Protocol for measurement and analysis of blood perfusion

For the measurements shown in Figure 1B, both legs were exposed, and the bilateral Zusanli acupoints (ST 36) were marked by senior acupuncture doctors. From the perspective of traditional acupuncture theory, ST 36 is closely related to the digestive system. However, in the current study, the aim was not related to traditional acupuncture theory, so we do not discuss the specificity of ST 36, which was simply used as an observation point for the lower extremities. Blood perfusion signals were recorded using a PeriFlux System 5,000 (Perimed AB, Stockholm, Sweden) with a 64 Hz sample rate and a 0.2s time constant. An optical fibre probe connected with a PeriFlux 5,000 was used to illuminate and collect the scattered light from the skin tissue. This technology mainly detects changes in microcirculation blood flow in the dermis rather than in deep muscle tissues. Attach the probe to the surface of interest with double-sided tape, and during the measurement process, the subjects remained in a static position was maintained constant to reduce the influence of external factors on the local blood flow. The recorded file of each subject was opened in PeriSoft (version 2.5.5, Perimed AB, Stockholm, Sweden) for Windows. The detailed data were exported in. txt format, imported into MATLAB (version 2021a) software and analysed. In the current dataset, data for blood perfusion on both sides were obtained simultaneously, and coherence analysis was performed (Wang et al., 2013; Wang et al., 2017). We define the frequency value corresponding to the peak value of blood flow coherence in the frequency range of 1.0–1.5 as the PF.

### 2.5 Electrogastrogram measurement protocol and analysis

The subjects maintained a supine position for measurements. The placement positions of the 8-channel gastrointestinal electrodes are shown in Figure 1C, where the first channel is used for electrocardiogram (ECG) data acquisition. The EGG recordings were processed with a NeurOne system (NeurOne, MEGA Electronics Ltd, Finland). The data were digitized with a sampling rate of 10,000 Hz and then down-sampled at 1000 Hz. EGG data were analysed using the FieldTrip toolbox (Oostenveld et al., 2011), and the analysis code for EGG analysis was provided by Wolpert et al. (https://github.com/niwolpert/EGG_Scripts) (Wolpert et al., 2020). For each channel, Fast Fourier Transform (FFT) algorithm was implemented in the Fieldtrip toolbox with a Hanning taper to estimate the spectral power of EGG. The largest activity in the 0.033–0.066 Hz (2–4cpm) was used to determine the dominant frequency (DF) of each channel. FFT was carried out on all channels, and eight wave peaks were obtained. The frequency corresponding to the maximum wave peak value represented the DF of each subject (Jia et al., 2023).

### 2.6 PNS index (PNSi) and SNS index (SNSi) analysis

In the process of ECG recording, we set the lead according to the electrode placement scheme in the previous study (Pietraszek and Komorowski, 2009), and the lead 1 can clearly detect the obvious QRS wave group. After exporting the original signal of lead 1 in European Data Format (EDF), it was imported into Kubios HRV software (Kubios Oy, Kuopio, Finland) for HRV analysis. The specific analysis process referred to the research methods of predecessors (Niskanen et al., 2004; Tarvainen et al., 2014; Cilhoroz et al., 2020):

The data were detrended using the smoothness prior approach with a lambda value of 500, and artefacts were corrected by applying the medium filter provided by Kubios HRV (Jia et al., 2023). The PNSi is calculated by the mean RR interval, the root mean square of successive differences (RMSSD) and the Poincaré plot (SD_1_) in normalized units. The SNSi is calculated by the mean HR, Baevsky’s stress index (SI) and Poincaré plot (SD_2_) in normalized units. SD_1_ mainly reflects the PNS regulation, and SD_2_ mainly reflects SNS regulation. The ratio of SD_1_/SD_2_ can further evaluate the balance between them (Vanderlei et al., 2009). Each parameter is standardized against normal population values and scaled by the standard deviations of these norms. A proprietary weighting process, considering the relationships between exercise intensity, heart rate, and HRV, is applied to compute the SNSi(Migovich et al., 2022).

### 2.7 Establishment and verification of the linear regression equation

The fitlm function built in MATLAB software (version 2021a) was used for linear regression analysis of the PF value and PNSi/SNSi value. To ensure the reliability of the experimental results, we used an external dataset to verify the PF-PNSi/SNSi regression model constructed. The dataset is from a published study (Jia et al., 2023), which can be downloaded from the Figshare platform to obtain open data (https://doi.org/10.6084/m9.figshare.14863581.v2). The PF values in this dataset were used to predict the PNSi and SNSi values. A paired t-test was used to determine whether there was a significant difference between the true and predicted values of PNSi/SNSi for each sample.

### 2.8 Statistical analysis

The data are presented as the mean ± SD and SD calculation is based on the original sample data. A paired t-test was used to compare pre- and post- stimulation data. Analysis of variance (ANOVA) was used to analyse between-subject factors. The correlations between the PNSi or SNSi and PF were analysed using SCCs. All correlation analyses were conducted using MATLAB software (version 2021a). All reported *P* values were two-sided and were corrected using the FDR method via the fdr tool package (Klaus and Strimmer, 2024). The corrected *P* value is represented as *P*_corr_. The level of significance was defined as *P* < 0.05.

## 3 Results

### 3.1 Participants

In this study, a total of 60 subjects were recruited, and all the subjects were included in the final statistical analysis. Detailed information on the subjects is summarized in Table 1.

**TABLE 1:** Sex, age, height and weight distributions.

Group	n	Sex (female/male)	Age (years, mean ± SD)	Height (cm, mean ± SD)	Weight (kg, mean ± SD)
4 °C	20	17/3	27 ± 3	165.0 ± 7.2	59.6 ± 9.3
10 °C	20	18/2	25 ± 3	163.6 ± 5.6	56.2 ± 10.7
30 °C	20	17/3	26 ± 1	161.9 ± 6.1	54.6 ± 13.6

### 3.2 EGG results

Gastric slow wave is the basic rhythmic electrical activity of gastric smooth muscle contraction, which determines the frequency and pattern of gastric wall contraction. Among these slow-wave activities, DF refers to the oscillation frequency that dominates the slow-wave electrical activity of the stomach, usually reflecting the rhythmic electrical activity in the gastric pacing area. DF is regulated by the pacing potential generated by Cajal interstitial cells (ICC) (Langton et al., 1989), coordinating the periodic contraction of gastric smooth muscle and driving the mixing and emptying of gastric contents (Ward et al., 2000). The stability of DF depends on the ICC network (Sanders et al., 2023), autonomic nerve regulation (Ward and Sanders, 2006) and hormonal environment, etc. In this study, after drinking glucose, the DFs of the subjects showed significant changes, mainly decreasing (Supplementary result S1). Referring to previous studies (Drossman, 2006; Wolpert, 2021), we think that the changes in gastric electrical activity might be related to the imbalance of autonomic nerve function.

### 3.3 PNSi and SNSi

**FIGURE 2:**
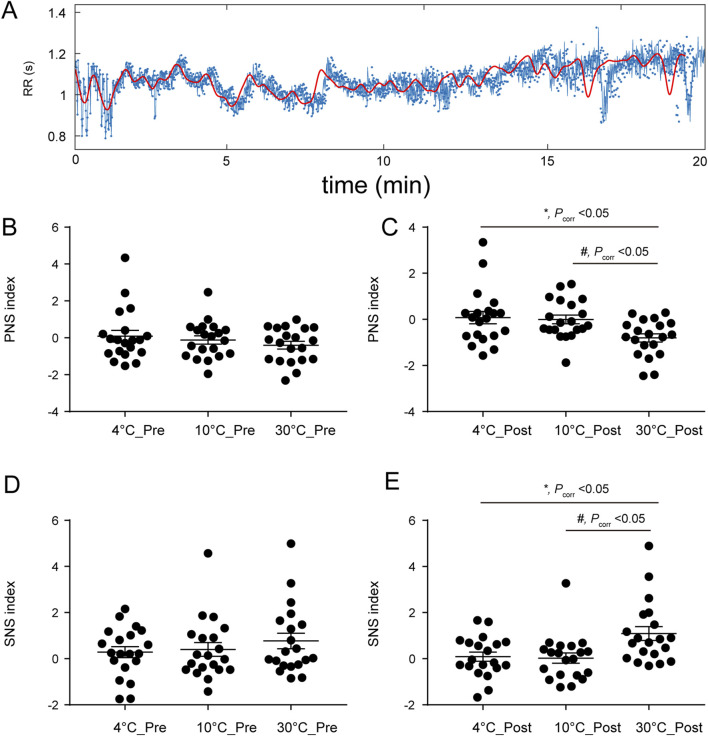
**(A)** RR interval of the ECG in channel 1; **(B)** Pre-stimulus PNSi, *F*_(2, 57)_ = 0.93, *P* > 0.05; **(C)** After stimulation, *F*_(2, 57)_ = 5.16, *P*_corr_<0.05; **(D)** Pre-stimulus SNSi, *F*_(2, 57)_ = 0.74, *P* > 0.05; **(E)** After stimulation, *F*_(2, 57)_ = 5.98, *P*_corr_<0.05. *P*_corr_: corrected *P* value.

The responses of the PNSi and SNSi to stimuli at different temperatures are shown in Figure 2. Figure 2A shows the 1-channel ECG RR interval. The PNSi of the different temperature groups (4 °C, 10 °C, and 30 °C) before stimulation are shown in Figure 2B. Before stimulation, there was not significantly different among the different temperature groups (*F*_(2, 57)_ = 0.93, *P* > 0.05). As shown in Figure 2C, there was a significant difference at different temperatures (*F*_(2, 57)_ = 5.16, *P*_corr_<0.05) after stimulation. The SNSi before stimulation is shown in Figure 2D (*F*_(2, 57)_ = 0.74, *P* > 0.05). After stimulation, as shown in Figure 2E, the SNSi changed significantly (*F*_(2, 57)_ = 5.98, *P*_corr_<0.05).

### 3.4 Correlation between PF and SNSi/PNSi

**FIGURE 3:**
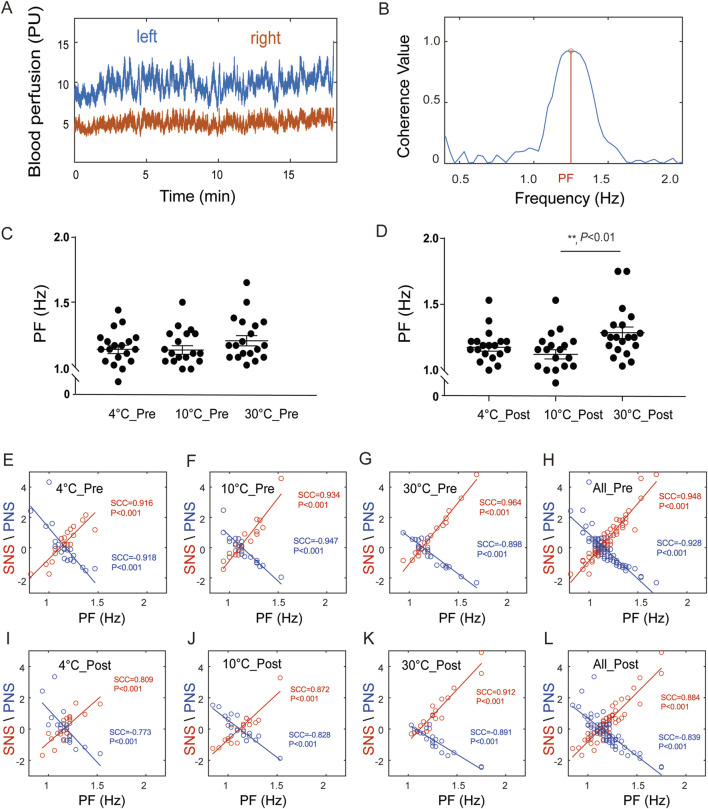
The correlations between the PNSi/SNSi and PF. **(A)** Blood perfusion levels on both sides; **(B)** Diagram of PF; **(C)** PF before stimulation, *F*_(2, 57)_ = 1.3, *P* > 0.05; **(D)** PF after stimulation, *F*_(2, 57)_ = 5.36, *P* = 0.0074; **(E–H)** Correlation analysis results before stimulation at 4 °C group, 10 °C group, 30 °C group, and all groups, respectively; **(I–L)** Correlation analysis results after stimulation at 4 °C group, 10 °C group, 30 °C group, and all groups, respectively.

The results of the bilateral blood perfusion consistency analysis are shown in Supplementary Figure S2. The blood perfusion are shown in Supplementary Figures S2A,S2B, respectively. As shown in Supplementary Figure S2C, the coherence curves of the blood flow signals of both lower limbs tended to be similar at different temperatures before stimulation. As shown in Figure 2D, after stimulation, the trend of the coherence curves between the groups at 4 °C and 30 °C and between 10 °C and 30 °C significantly differed, with obvious peaks at some frequencies, and the grey shadow area reflects that the frequency window fell into significant clusters. Then the correlation between PF and PNSi or/SNSi was analysed through Spearman correlation coefficient (SCC) (Supplementary Methods S1), the results are shown in Figure 3. The raw data of both side blood perfusion are shown in Figure 3A The PF, which corresponding to the peaks in the 1.0–1.5 frequency range, as shown in Figure 3B As shown in Figure 3C, there was no significant difference in PF values (*F*_(2, 57)_ = 1.3, *P* > 0.05). After stimulation, the PF significantly differed (*F*_(2, 57)_ = 5.36, *P* = 0.0074) in different groups (as shown in Figure 3D). The correlation between the PNSi/SNSi and the PF value are shown in Figures 3E–L. The results indicate that whether before (Figures 3E–H) or after stimulation (Figures 3I–L), there is a significant positive correlation between SNS and PF, while there is a significant negative correlation between PNS and PF. To ensure the universality and reliability of the results, we conducted additional control experiments and found that even under normal saline conditions, the significant correlation between PF and SNS/PNS remained stable (Supplementary Material Result S3).

### 3.5 Linear regression and validation

**FIGURE 4:**
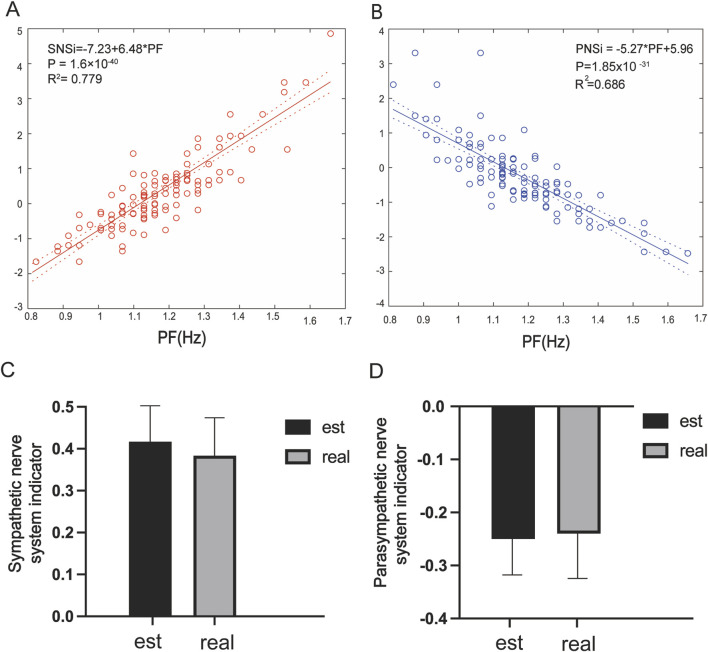
**(A)** Linear regression analysis of PF and the SNSi, *β =* 6.48, *R* ^*2*^ *=* 0.779*, P* = 1.6 × 10^−40^; **(B)** Linear regression analysis of PF and the PNSi, *β =* −5.27*, R* ^*2*^ *=* 0.686*, P =* 1.85 × 10^−31^; **(C)** Estimated vs. real SNSi in the dataset; **(D)** Estimated vs. real PNSi in the dataset.

The quantitative relationship between PF and the SNSi/PNSi is shown in Figure 4. Indicating that there is a real linear relationship between the PF and SNSi, and the regression relationship is extremely significant. The established linear regression equation *SNSi* = 6.48 *PF-7.23 is statistically significant (Figure 4A). For PNSi, the established equation *PNSi* = −5.27 *PF+5.96 is also statistically significant (Figure 4B). To further verify the reliability of the established equation, an independent dataset was used to predict the SNSi (Figure 4C) and PNSi (Figure 4D) through PF values. The results show that there is no difference between the real values and the estimated values, whether it is SNSi or PNSi.

## 4 Discussion

In the present study, we present a new method for assessing ANS function on the basis of peripheral blood flow signal analysis. This randomized controlled study included healthy adults with an average age of 26 years, and we observed a significant response in blood perfusion of the lower extremities after they drank glucose at different temperatures. These findings indicate that temperature stimulation significantly affects gastric electrical activity, HRV and coherence of blood flow in both lower limbs. More significantly, to our knowledge, this is the first time that a significant correlation has been reported between the PF of blood flow and the PNSi/SNSi of HRV. This correlation suggests that peripheral blood flow signals, particularly PF, may serve as potential indicators of ANS function, suggesting a noninvasive assessment method that could complement conventional HRV analysis.

### 4.1 ECG and EGG changes due to glucose stimulation by drinking water at different temperatures

The results of the present study revealed that, regardless of temperature stimulation, there was some effect on the main power frequency of the EGG. In the absence of stimulation, no significant differences were observed in the PNSi/SNSi across the 3 temperature groups, suggesting baseline equivalence in ANS activity. However, after stimulation, a significant difference in the PNSi/SNSi was found between the groups, indicating that the ANS is sensitive to stimulation by temperature changes in the digestive system.

A previous study suggested that water intake activates different gastrointestinal vagal afferents in a temperature-dependent manner and may affect cardiac vagal tone (Girona et al., 2014). Given the sensitivity of the digestive system to temperature changes, it is conceivable that such changes could directly affect autonomic functional control, not only in the digestive tract but also throughout the body (Morris et al., 2017; Sarafian et al., 2018). Our study builds on these findings by demonstrating that the ingestion of glucose at different temperatures induces changes in cutaneous vasomotor activity, implying that cutaneous vasomotor modulation may play an important role in the digestive process, especially in response to changes in the temperature of the ingested fluid.

The rationale for selecting glucose solutions over water in our study is multifaceted. Previous studies have shown that drinking water increases muscle sympathetic activity and subtle changes in heart rate and blood pressure in healthy subjects in the absence of a pressor response (Scott et al., 2001). These sympathetically mediated responses, such as decreased heart rate, increased total peripheral resistance, and increased baroreceptor sensitivity, are not reliant on gastrointestinal stretch but are instead associated with the osmolality of the ingested fluid. This osmotic effect may trigger the body’s autonomic cardiovascular response through osmolarity-sensitive nerve fibres within the intestinal or portal circulation (Brown et al., 2005; May and Jordan, 2011). To reduce the potential stimulation of osmoreceptors, in the present study, we opted for isotonic solutions, which maintain a consistent osmotic pressure across different temperatures, thus allowing us to isolate the effects of temperature from those of osmolality on sympathetic tone. The choice of a 5% glucose solution over saline in this study further addresses the potential interactions between osmotic stimuli and sodium ions, considering the impact of sodium on vagal function and its role in cardiovascular regulation (Stocker et al., 2024).

### 4.2 Quantitative methods for analysing SkBF signals

According to our hypothesis, SkBF signals, which are subject to alterations due to ANS activity, necessitate a robust quantitative analysis method for accurate assessment. Coherence analysis is a well-established quantitative method that has been widely used in biosignal analysis to assess the relationship between two signals (Bowyer, 2016). This method is characterized by its usefulness for measuring the consistency, or synchronization, between two time series datasets at different frequency bands. Conventionally, coherence analysis has been employed to analyse the consistency of signals in ECGs and EMGs (Siemionow et al., 2010; Busonera et al., 2018), but its application extends to the analysis of the complex dynamics of the cardiovascular system (Tankanag et al., 2014; Ticcinelli et al., 2017). It can identify frequency-specific correlations within SkBF signals and effectively evaluate the synchronization of two time series at different frequencies, which is crucial for understanding the dynamic process of blood flow regulation. In this context, it is used to determine the synchronization between peripheral blood flow signals and how these signals respond to specific stimuli (e.g., temperature changes).

In this study, we employed coherence analysis to assess the synchronization between bilateral peripheral blood flow signals. These findings suggest that temperature stimulation has distinct effects on the synchronization of blood flow signals across different frequency bands, potentially related to the regulatory mechanisms of the ANS.

By applying coherence analysis to our SkBF data, we can quantify the impact of temperature-induced changes on blood flow consistency, thereby providing a more detailed assessment of ANS function. The coherence value, a quantitative metric ranging between 0 and 1, can objectively describe the strength of the relationship between two signals. A high coherence value suggests that the signals may be regulated by a common physiological mechanism, such as ANS activity, whereas a low coherence value may indicate a lack of consistency between signals or the influence of noise.

### 4.3 Potential of SkBF signals for assessing ANS function

In the biomedical field, the assessment of ANS activity is commonly used in methods such as HRV, which primarily reflects cardiac autonomic regulation (Shaffer and Ginsberg, 2017). In fact, HRV reflects more changes in cardiac autonomic regulation (ANS cardiovascular branch) and electrophysiological indicators (Carandina et al., 2021). The current framework associating the frequency components of HRV (low-frequency, LF and high-frequency, HF) with the divisions of the ANS (sympathetic and parasympathetic) has been considered too reductionist (Hayano and Yuda, 2019). Our study introduces a novel perspective by examining SkBF signals as potential indicators of ANS function. These signals, which reflect the integrated activity of the cardiovascular, respiratory, and digestive systems, offer a more comprehensive view of ANS activity beyond the cardiac domain.

A key innovation in our approach is the introduction of the PF value, defined as the frequency at which the coherence value peaks within the 1.0–1.5 Hz range, as derived from coherence analysis. This metric captures the synchrony of blood flow fluctuations in the lower limbs and serves as an indicator of ANS-mediated vascular control. The selection of the 1.0–1.5 Hz range is based on our preliminary findings that this frequency band corresponds to significant changes in blood flow in response to digestive temperature stimulation.

Unlike previous researchers (Jia et al., 2023), we are the first to discover a significant correlation between the PF value and the SNSi/PNSi. The results show that PF values are sensitive to changes in blood flow induced by changes in temperature. This sensitivity suggests that PF values have the potential to serve as indicators of ANS function, reflecting the dynamic response of this system to stimulation. The correlation between PF values and the HRV indices assessed in this study further demonstrates the feasibility and validity of using peripheral blood flow signals to quantitatively assess ANS activity.

While the PF value, as we have introduced, is derived from a single characteristic point within the frequency domain, it represents a significant advancement in our ability to assess ANS function. The significant correlations we observed for this sole feature highlight the potential for further exploration within the broader frequency spectrum. This suggests that there is ample room for future research to uncover additional insights by examining other frequency bands or by employing more nuanced analyses of blood flow signals. Our findings pave the way for more detailed studies that could leverage the full spectrum of information contained within peripheral blood flow signals to provide an even more comprehensive understanding of ANS activity.

### 4.4 Strengths and limitations

This study demonstrates the potential of bilateral peripheral blood flow signal analysis as a novel method for assessing ANS function.

Despite these promising findings, our study has several limitations that warrant further investigation. First, the study sample consisted mainly of young and female participants, and it is not known whether the results would differ across broader populations, including different age groups and males. Future studies should aim to include a more diverse range of participants to increase the robustness of the findings. Second, the sample size in this study was relatively small, which might have influenced the statistical power and generalizability of the results. Increasing the sample size in future research could strengthen the validity of the findings and provide more reliable conclusions. Additionally, the current study focused on a limited recording method, specifically analysing blood perfusion signals at two specific points. A more comprehensive assessment of autonomic function may be possible if the recording is extended to more regions of the body and includes a wider range of physiological parameters. Furthermore, the analyses were performed on the basis of specific frequency bands and their peaks, and future research could explore more advanced feature extraction techniques to capture a wider range of information in the blood flow signals.

## 5 Conclusion

This study provides valuable insights into the potential of analysing bilateral peripheral blood flow signals to assess ANS function and is expected to be a noninvasive and comprehensive approach to assess autonomic regulation.

## Data Availability

The original contributions presented in the study are publicly available. This data can be found here: Figshare:https://doi.org/10.6084/m9.figshare.28636658.v2. Further inquiries can be directed to the corresponding authors.

## Abbreviations

SkBF, skin blood flow; FFT, Fast Fourier Transform; DF, dominant frequency; PF, The frequency value corresponding to the peak coherence of blood flow within the frequency range of 1.0–1.5; RMSSD, root mean square of successive differences; ST 36, Zusanli acupoint; ECG, electrocardiogram; EGG, electrogastrogram; HRV, heart rate variability.
