# Supplementary material for: Coherence analysis of peripheral blood flow signals is a potential method for evaluating autonomic nervous system function
Source: Front Physiol. 2025 Oct 13;16:1658174. doi: 10.3389/fphys.2025.1658174 (PMC12554719; doi:10.3389/fphys.2025.1658174)
Supplement: Supplementary file 1 [file Supplementaryfile1.docx]

Coherence analysis of peripheral blood flow signals is a potential method for evaluating autonomic nervous system function

**Qiuyue Lyu^1^†, Yuning Qin^3^†, Xin Wang^4^†, Qizhen Wang^3^, Qi Liu^1,2^, Na Tu^1,2^, Yuhan Liu^1,2^, Zixin Huo^1,2^，Xiaojing Song^1^, Shuyou Wang^1^, Weibo Zhang^1^, Xue Cao^3^, Enshi Lu^3^, Xiaoliang Zhao^3^, Shuyong Jia^1^*,Liyun He^3^*, Guangjun Wang^1^***

1. Institute of Acupuncture and Moxibustion, China Academy of Chinese Medical Sciences, Beijing, China
2. Tianjin University of Traditional Chinese Medicine, Tianjin, China
3. Institute of Basic Research in Clinical Medicine, China Academy of Chinese Medical Sciences, Beijing, China
4. Chinese Medicine Data Center, China Academy of Chinese Medicine, Beijing, China

Qiuyue Lyu: lyuqy@itcm.ac.cn

Yuning Qin: yuning221@163.com

Xin Wang: 493630907@qq.com

Qizhen Wang: 18600759031@163.com

Qi Liu: 17803899217@163.com

Na Tu:18073091680@163.com

Yuhan Liu: a974423953@163.com

Zixin Huo:huo202403@163.com

Xiaojing Song: xts2010@163.com

Shuyou Wang: wangsy15@126.com

Weibo Zhang: zhangweibo@hotmail.com

Xue Cao: cxcy511@163.com

Enshi Lu: luenshi1996@163.com

Xiaoliang Zhao: [772956914@qq.com](mailto:772956914@qq.com)

†: contributed equally

*Corresponding author:Shuyong Jia(shuyong6666@163.com);

Liyun He(hely3699@163.com);

Guangjun [Wang(wangguangjun@mail.cintcm.ac.cn)](mailto:Wang(wangguangjun@mail.cintcm.ac.cn))

**Methods**

**S1.Correlation analysis**

We define the frequency value corresponding to the peak value of blood flow coherence in the frequency range of 1.0-1.5 as the PF. The correlations between PF and HRV PNSi or/ SNSi were analysed via Spearman’s correlation coefficient (*SCC*), which was estimated by the following equation:

$$SCC(X,Y)= \frac{\sum_{i} (x_{i}-\bar{x})(y_{i}-\bar{y})}{\sqrt{\sum_{i} {(x_{i}-\bar{x})}^{2}{(y_{i}-\bar{y})}^{2}}}$$

In this study, $X$ represents the PF set of all samples, $Y$ represents the HRV, PNSi, or SNSi set of all samples, $x_{i}$ represents the PF rank of the $i$th sample in all samples, and $y_{i}$ represents the HRV PNSi or SNSi rank of the $i$th sample in all samples.

To determine the frequency dynamics of coherence responses to different stimuli, cluster-based permutation tests were carried out to identify clusters where a significant difference in coherence value was present for different stimulation conditions on the basis of the specific frequency intervals of interest. If the calculated P value was less than the critical alpha level of 0.05, then the clusters of differences between different conditions were considered significant52. This was implemented using the source code provided by Edden M. Gerber (https://www.mathworks.com/matlabcentral/fileexchange/71737-permutest).

**Results**

**S1.EGG results**

The results of the EGG analysis after stimulation at different temperatures are shown in Fig. S1. The spectral analysis of each signal before stimulation is shown in Fig. S1A, and the spectral analysis of each signal after stimulation is shown in Fig. S1B,which revealed that there is a significant change in the frequency domain after stimulation, and the number of peaks in the subjects changes from a single peak to a double peak and then shifts to both sides. After stimulation, the DFs of the subjects changed significantly, mainly decreasing. As shown in Fig. S1C, the 4°C group, significant changes were observed (*P*<0.01), with a significant decrease in frequency at peak power, indicating a significant effect of temperature on EGG activity. Similarly, the 10°C group also showed significant changes(Fig. S1D), whereas the 30°C group did not (Fig. S1E).


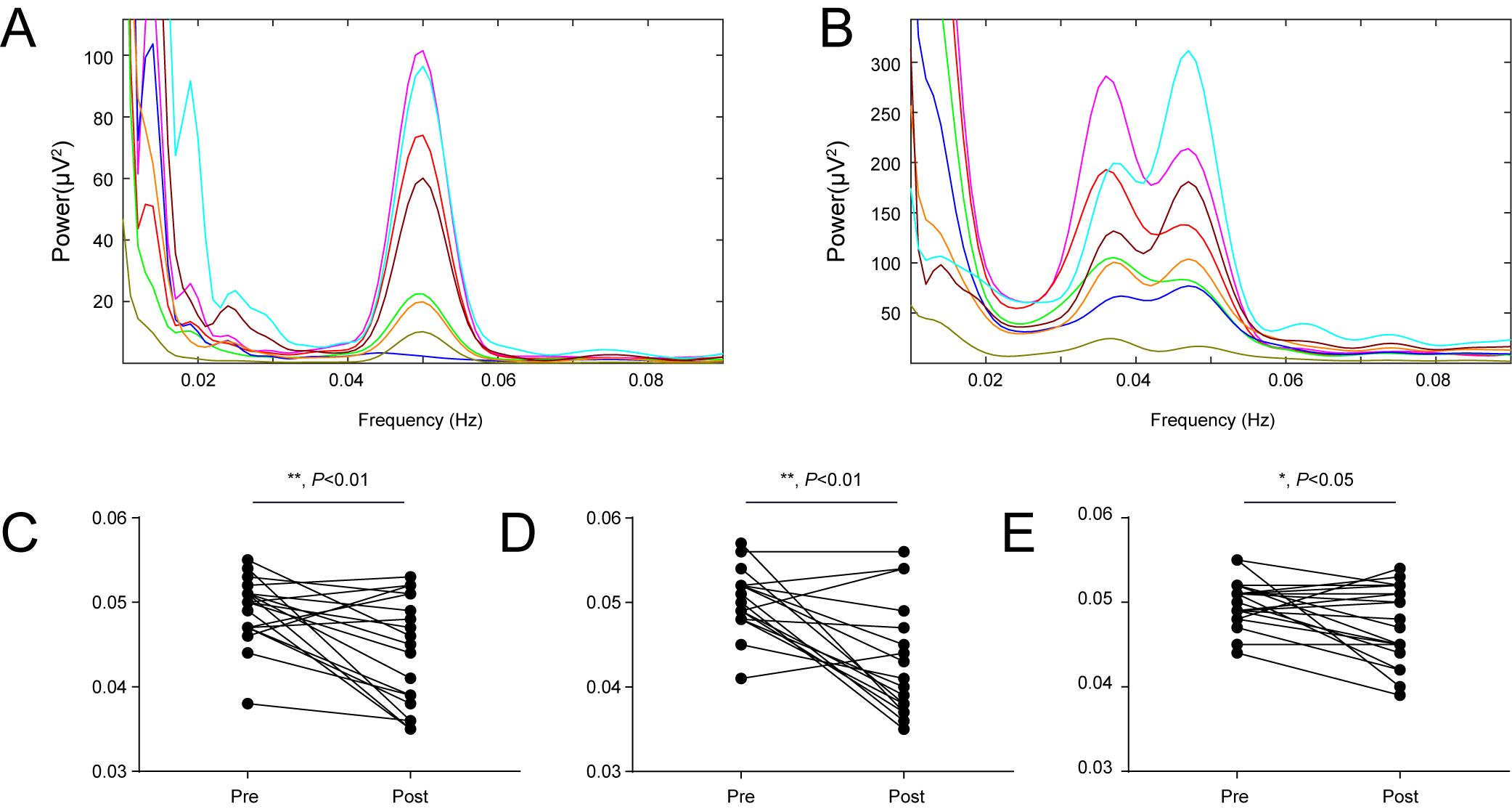


Supplementary Fig. S1. EGG power spectra changes. (A) EGG peak frequency before stimulation; (B) EGG peak frequency b after stimulation; (C) frequency corresponding to the DF value before and after stimulation in the 4°C group, *P*<0.01 (4°C); (D) frequency corresponding to the DF value before and after stimulation in the 10°C group, *P*<0.01 (10°C); (E) frequency corresponding to the DF value before and after stimulation in the 30°C group, *P*<0.05(30°C). EGG, electrogastrogram; DF: dominant frequency.

**S2.Blood perfusion**


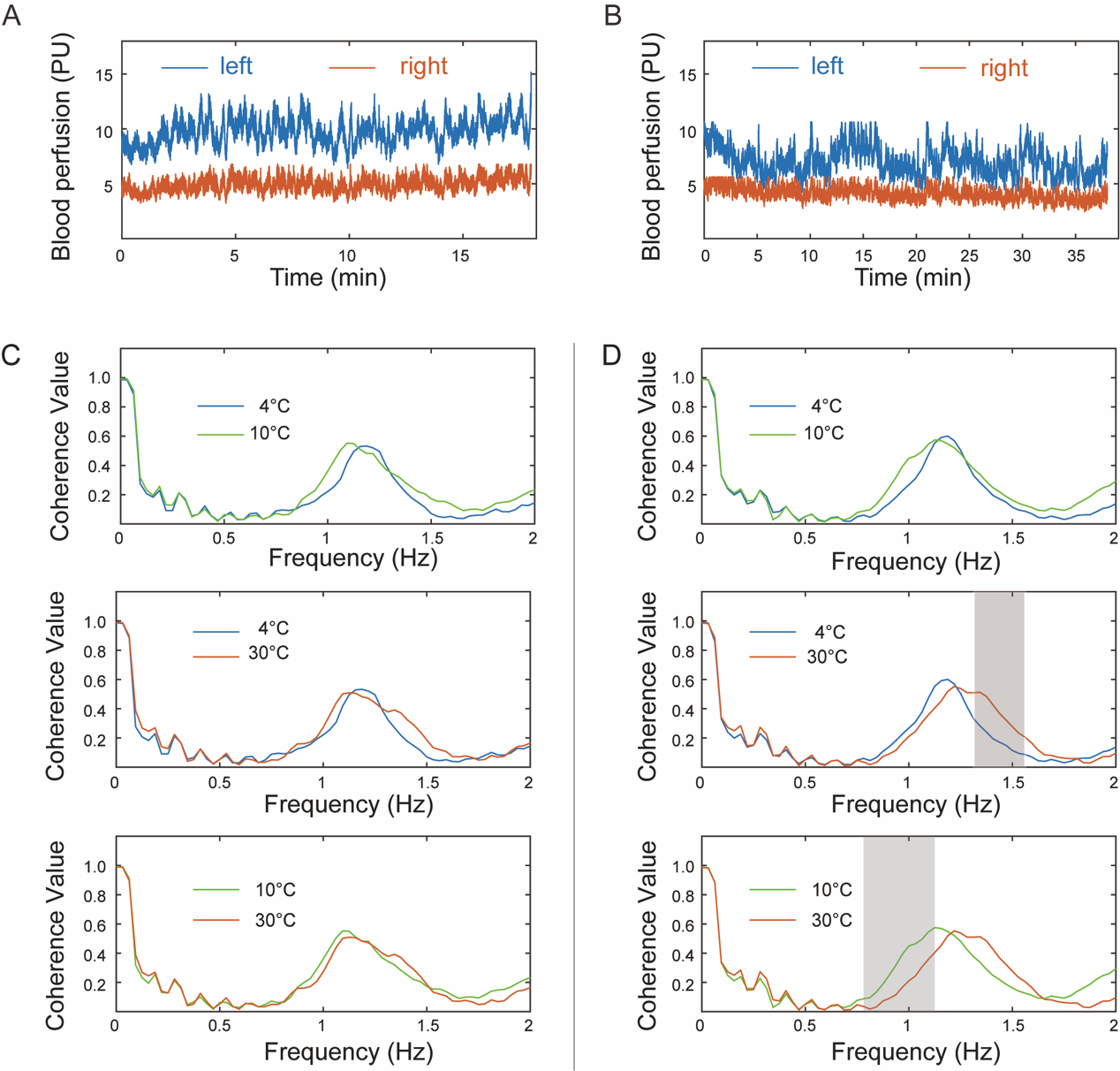
Supplementary Fig. S2. Results of bilateral blood perfusion consistency analysis. (A) Before stimulation; (B) After stimulation; (C) Changes in bilateral skin blood flow coherence values with frequency before stimulation in different temperature groups; (D) Changes in the coherence value of bilateral skin blood flow with frequency after stimulation in subjects with different temperatures. The grey shaded areas reflect frequency windows that fall into significant clusters.

**S3 Correlation between PF and SNSi/PNSi（Saline）**

The correlation between the PNSi/SNSi and the PF value are shown in Fig. S3A-F. The results indicate that whether before (Fig. S3A, B,and C) or after stimulation (Fig. S3D, E, and F), there is a significant positive correlation between SNS and PF, while there is a significant negative correlation between PNS and PF.

Supplementary Fig. S3.The correlations between the PNSi/SNSi and PF. (A)–(C) Correlation analysis results before stimulation at 4°C group, 10°C group and 30°C group, respectively; (D)–(F) Correlation analysis results after stimulation at 4°C group, 10°C group and 30°C groups, respectively.
